# Sub-fossil beetle assemblages associated with the “mammoth fauna” in the Late Pleistocene localities of the Ural Mountains and West Siberia

**DOI:** 10.3897/zookeys.100.1524

**Published:** 2011-05-20

**Authors:** Evgeniy Zinovyev

**Affiliations:** Institute of Ecology of Plants and Animals, Ural’ Branch of the RAS, Ekaterinburg, Russia

**Keywords:** Carabidae, Coleoptera, sub-fossil beetles, fauna change, insect assemblages

## Abstract

The distribution of beetles at the end of the Middle Pleninglacial (=terminal Quaternary) was examined based on sub-fossil material from the Ural Mountains and Western Siberia, Russia. All relevant localities of fossil insects have similar radiocarbon dates, ranging between 33,000 and 22,000 C14 years ago. Being situated across the vast territory from the southern Ural Mountains in the South to the middle Yamal Peninsula in the North, they allow latitudinal changes in beetle assemblages of that time to be traced. These beetles lived simultaneously with mammals of the so-called “mammoth fauna” with mammoth, bison, and wooly rhinoceros, the often co-occurring mega-mammalian bones at some of the sites being evidence of this. The beetle assemblages found between 59° and 57°N appear to be the most interesting. Their bulk is referred to as a “mixed” type, one which includes a characteristic combination of arcto-boreal, boreal, steppe and polyzonal species showing no analogues among recent insect complexes. These peculiar faunas seem to have represented a particular zonal type, which disappeared since the end of the Last Glaciation to arrive here with the extinction of the mammoth biota. In contrast, on the sites lying north of 60°N, the beetle communities were similar to modern sub-arctic and arctic faunas, yet with the participation of some sub-boreal steppe components, such as *Poecilus ravus* Lutshnik and *Carabus sibiricus* Fischer-Waldheim. This information, when compared with our knowledge of synchronous insect faunas from other regions of northern Eurasia, suggests that the former distribution of beetles in this region could be accounted for both by palaeo-environmental conditions and the impact of grazing by large ruminant mammals across the so-called “mammoth savannas”.

## Introduction

One of the main tasks of any zoological investigation is the study of the influence of environmental factors on the structure of communities, including changes in insect faunas. These changes may be estimated from modern faunas. But it is necessary to study such factors, which could define the specific structure of insect communities in the past.

With respect to research on palaeo-entomological processes, it is extremely difficult to estimate the character of external influences on the structure of communities, because there are no real opportunities to inspect them directly. It is only possible to make reconstructions, which are based on the analysis of sub-fossil insect assemblages found in Quaternary strata. The term “sub-fossil” means, that insect remains are presented in these layers by isolated chitin fragments not yet fossilized. Present ecological requirements of these species can be extrapolated to the period of the past investigated; the conclusions of which can be compared with results of palaeo-botanical analysis and studies of mega and small mammals. The comparison of these conclusions allows a reconstruction to be made of palaeoenvironmental conditions prevailing in the given territory in the analyzed period of the past.

The aim of this study is to try to explain peculiarities of the insect faunas in relation with the paleoenvironmental conditions of the terminal phase of the Late Pleistocene and estimate the factors possibly determining the composition of insect species in the past, including the influence of the large herbivorous mammals.

## Materials

To this end, I took some synchronous sites situated in the vast territory from the Jamal peninsula in the North up to vicinities of Ekaterinburg city in the South. Radiocarbon dating confirmed the synchrony of these sites. The period of investigations covers the end of the Late Pleistocene including terminal phase of Middle Pleninglacial period and the beginning of the Late Pleninglacial or Late Glacial Maximum (LGM). Chronologically this time corresponds to the end of Maritime Isotope Stage (MIS) 3 and the beginning of MIS 2; 33,000–22,000 years Before Present (BP). This period is considered by geologists as the most severe time of the Late Pleistocene and characterized by a cooler-than-present climate which fluctuated heavily on time scales of a few thousand years (Adams et al. 1999; Adams and Faure 1999; Arkhipov and Volkova 1994; Astakhov 2009; Bos et al. 2004).

**Figure 1.**
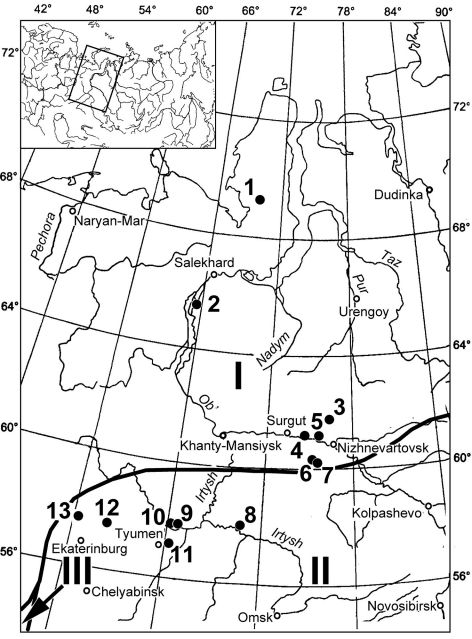
Geographical location of the study sites in the Ural Mountains and Western Siberia. Numbers of sites: **1** Syoyakha-Mutnaya **2** 430 km from Ob **3** Aganskiy uval-1290/2 **4** Mega **5** Lokosovo **6** Kul’egan-2247 Point I **7** Kul’egan -2247 Point II **8** Skorodum **9** Andriyshino **10** Nizhnyaya Tavda **11** Mal’kovo **12** Nikitino **13** Shurala. Bold line - Borders of vegetation types, reconstructed for the beginning of MIS 2 on the basis of palynological data: **I** periglacial tundra **II** periglacial steppe and forest-steppe **III** boreal forest and parklands (after Grichuk and Borisova 2009)

The work is based on sub-fossil material obtained from 13 sites scattered over the large territory of the Ural Mountains and West Siberia (Figure 1; Table 1). Sub-fossil insect remains were found in deposits exposed both in quarries and in river banks. Field sampling was done using the standard techniques in Kiselev (1987). Geologists provided geological descriptions of the sites and their provisional dating; most samples were radiocarbon-dated (Table 1). Laboratory treatment and the subsequent identification of fossil specimens were performed at the Institute of Plant and Animal Ecology in Ekaterinburg. The classification of the sub-fossil insect faunas used is that proposed by the author (Zinovyev 2006).

**Table 1.** Chronological position of the study sites dated by the end of the Middle Pleninglacial period and associated with the “mammoth fauna”

Sites where sub-fossil insect faunas were found			Coordinates		Radiocarbon data, years before present (yr. BP). The abbreviation of organization and laboratory number of this data are given in parenthesis
						N	E
1.	Syoyakha-Mutnaya (V.I.Nazarov, unpublished data)		72°25'		66°48'	30,700±1100 (UPI-716)
2.	430 km of Ob		64°25'	70°50'	24,000±1500 (IPAE-63)
3.	Aganskiy uval-1290/2		61°22'	76°45'	23,300±575 (IPAE-95)
4.	Mega-2172		60°56'	72°20'	33,100±2300 (IOAN 132)
			60°56'	72°20'	26,285±590 (SOAN 982)
5.	Lokosovo		60°40'	71°32'	22,930±650 (SOAN 956)
6.	Kul’egan-2247 Point I		60°25'	75°50'	21,815±225 (SOAN-6837)
7.	Kul’egan -2247 Point II		60°25'	75°50'	26,730±250 (LOIA-8663)
8.	Skorodum		57°47'	70°58'	26,500±550 (SOAN 4538)
9.	Andriyshino		57°41'	66°08'	approximately 30,000 C14 yr. BP
10.	Nizhnyaya Tavda		57°41'	66°12'	27,400±335 (SOAN 4534)
			57°41'	66°12'	24,820±750 (SOAN 4535)
11.	Mal’kovo		56°25'	66°10'	31,800±350 (GIN-5338)
12.	Nikitino		57°34'	63°17'	24,480±550 (SOAN 4537)
			57°34'	63°17'	28,460±800 (SOAN 4536)
13.	Shurala	Plant detritus	57°28'	60°15'	27,600±150 (Ki-15505)
		Mammalian bone	57°28'	60°15'	36,700±250 (Ki-15512)

## Results

All studied insect faunas occurred in the interval between 33,000 and 22,000 C14 yr. BP (Table 1), the terminal phase of the Middle Pleninglacial (MIS 3). These studies cover the vast territory between 67° and 57°N. I tried to trace elements of latitudinal zonality and estimate factors affecting natural ecosystems and insect faunas.

According to the classification by Zinovyev (2006), the studied faunas can be referred to as arctic, sub-arctic, "mixed" and boreal types.

Only faunas of the arctic type were found at the sites lying north of 61°N latitude (sites 1–3 in Table 1). The main characteristics of these faunas are:

1. Dominance or sub-dominance of arctic species – *Curtonotus alpinus*, *Pterostichus costatus*, *Pterostichus sublaevis* and the rove beetle *Tachinus cf. arcticus* (Table 2).

2. Dominance or sub-dominance of sub-arctic species of the sub-genus *Cryobius* and the species *Pterostichus pinguedineus*, *Pterostichus ventricosus*, *Diacheila polita*, *Curtonotus torridus* (Table 2).

3. Single occurrences of sub-boreal steppe species – *Carabus sibiricus*, leaf beetles *Chrysolina perforata*, *Chrysolina aeruginosa*. Only one elytrum of a specimen of *Poecilus ravus* was found in the Aganskyi uval-1290/2 site (61°22'N, 76°45'E).

Entomo-complexes referred to as “arctic” allow the reconstruction of severe environmental conditions similar to the modern arctic tundra, characterized by a cold climate with temperatures of July +12°C, January -27°C, the distribution of open landscapes and the absence of wood.

Between 61° and 59°N, the fossil beetle faunas of the sub-arctic type are similar to the recent communities of the south tundra and forest tundra (sites 4–7 in Table 1). The main characteristics of these faunas are:

1. Presence of arctic species – *Curtonotus alpinus*, *Pterostichus costatus* and the rove beetle *Tachinus cf. arcticus* (but in fewer quantities than in arctic faunas).

2. Dominance of sub-arctic species presented by the sub-genus *Cryobius* of genus *Pterostichus*, *Pterostichus pinguedineus*, *Curtonotus torridus*, *Diacheila polita* (Table 2).

3. Occurrence of sub-boreal steppe species – *Carabus sibiricus*, *Poecilus ravus*, the weevil *Stephanocleonus eruditus*, and the carrion beetle *Aclypaea sericea*.

4. Presence of single xylophagous beetles associated with larch or spruce, the weevil *Callirus albosparsus*, and the bark beetle *Phoelotribus spinulosus*.

Insect assemblages referred to as belonging to the “sub-arctic” type, are similar to modern insect faunas from the southern part of the contemporary Sub-arctic. Presumably, reconstructed landscapes look like modern south tundra or forest tundra with the presence of single trees, such as larch or spruce. The thermal regime is probably characterized by several temperatures: July +13° – +14°C, January -25° – -26°C. These reconstructions are confirmed by palaeo-botanical data.

The faunas from sites situated south of 59°N are of a “mixed” type characterized by species combinations not presently found together; insect complexes of the majority of these localities resemble each other, with main features:

1. Dominance or sub-dominance of weevils *Otiorhynchus* similar to *Otiorhynchus politus*.

2. Presence of arctic and sub-arctic species – *Pterostichus (Cryobius)* spp., *Curtonotus alpinus*, the carrion beetle *Aclypaea sericea*.

3. Presence of sub-boreal steppe and sub-alpine insects – *Poecilus (Derus)* spp., *Cymindis mannerheimi*, *Pseudotaphoxenus dauricus*.

4. Occurrence of some halophylous beetles – *Pogonus* spp., darkling beetles *Belopus* spp.

5. Occurrence of xylophagous beetles (e.g., the bark beetle *Phoelotribus spinulosus*).

These faunas have no analogues among modern insect complexes, and may be classified as indicative of tundra steppe, although their species composition differs from that known from relict tundra steppe communities found today in Eastern Siberia and described by Berman (2001).

**Table 2.** Species of beetles found in the study sites associated with the “mammoth fauna”

Type of range*	*Taxon*	*Sites (see Table 1)*												
		*1*	*2*	*3*	*4*	*5*	*6*	*7*	*8*	*9*	*10*	*11*	*12*	*13*
	COLEOPTERA:													
	Carabidae:													
a-sb	*Carabus sibiricus* F.-W.						+	+					+	
a-sb	*Carabus cf. sibiricus* F.-W.		+											
	*Carabus (Trachycarabus)* sp.									+				
a	*Carabus truncaticollis* Esch.			+										
a	*Carabus cf. truncaticollis* Esch.		+											
sa	*Carabus cf. odoratus* F.-W.		+											
	*Carabus (Morphocarabus)* sp.							+						
	*Carabus* sp.		+	+					+	+			+	+
p	*Nebria rufescens* Sturm						+							
sa	*Nebria nivalis* Payk.							+	+					
	*Nebria* sp.	+												
sa	*Pelophila borealis* Payk.		+				+	+	+	+	+	+	+	
p	*Elaphrus riparius* L.		+											
p	*Notiophilus cf. aestuans* Motsch.													+
p	*Nebria cf. aquaticus* L.			+		+	+	+	+	+			+	
b	*Nebria reitteri* Spaeth								+		+			
b	*Nebria biguttatus* F.								+					
	*Nebria* sp.							+	+	+	+		+	
sa	*Blethisa catenaria* Brown.		+		+		+	+						
p	*Blethisa multipunctata* L.				+									
sa	*Diacheila polita* Fald.	+	+	+	+		+	+	+	+	+	+	+	
sa	*Diacheila arctica* Gyll.				+					+				
p	*Elaphrus riparius* L.				+			+	+				+	
sa	*Elaphrus lapponicus* Gyll.								+					
b	*Elaphrus angusticollis* R. F. Sahlb.						+	+	+					
b	*Elaphrus cf. angusticollis* R. F. Sahlb.								+					
	*Elaphrus* sp.						+			+				
p	*Lorocera pilicornis* F.													+
p	*Clivina fossor* L.							+		+				+
p	*Dyschiriodes cf. globosus* Hbst.													+
	*Dyschiriodes* sp.								+	+			+	
b	*Trechus secalis* Payk.										+			
b	*Trechus rivularis* Gyll.						+	+						
b	*Bembidion striatum* F.								+				+	
b	*Bembidion velox* L.								+					
sa	*Bembidion captivorum* Net.						+							
sa	*Bembidion scandicum* Lindr.						+							
b	*Bembidion ovale* Motsch.						+							
sa	*Bembidion umiatense* Lindr.								+					
sa	*Bembidion cf. umiatense* Lindr.							+	+	+				
b	*Bembidion infuscatum* Dej.									+	+			
b	*Bembidion cf. infuscatum* Dej.									+				
sa	*Bembidion grapei* Gyll.								+				+	
sa	*Bembidion cf. grapei* Gyll.							+		+				
b	*Bembidion scopulinum* Kby.												+	
b	*Bembidion deletum* Serv.								+					
b	*Bembidion cf. deletum* Serv.								+					
p	*Bembidion cf. tetracolum* Say									+				
	*Bembidion (Ocydromus)* sp.						+	+	+	+			+	+
sa	*Bembidion fellmanni* Mnnh.												+	
sa	*Bembidion cf. fellmanni* Mnnh.						+	+		+				
	*Bembidion (Plataphodes)* sp.							+	+				+	
p	*Bembidion obliquum* Ol.													+
	*Bembidion (Bembidionetolitzkya)* sp.												+	
	*Bembidion* sp.			+				+	+	+	+		+	+
sb	*Pogonus cf. punctatulus* Dej.												+	
sb	*Pogonus cf. cumanus* Lutschn.								+					
sb	*Pogonus cf. meridionalis* Dej.								+	+				
sb	*Pogonus cf. transfuga* Chaud.									+				
sb	*Pogonus* sp.								+	+		+	+	+
p	*Patrobus septentrionis* Dej.			+			+	+	+	+			+	
p	*Pogonus assimilis* Chd.								+				+	
sb	*Poecilus major* Motsch.										+			+
sb	*Pogonus cf. major* Motsch.									+		+		
sb	*Pogonus ravus* Lutshn.			+			+	+	+	+	+	+	+	
sb	*Pogonus cf. ravus* Lutshn.								+	+			+	
sb	*Pogonus hanhaicus* Tsch.									+				
sb	*Pogonus cf. hanhaicus* Tsch.								+	+			+	
sb	*Pogonus (Derus)* sp.								+	+				
t	*Pogonus lepidus* Leske								+					
	*Pogonus (s.str.)* sp.							+					+	
p	*Pterostichus nigrita* F.			+			+							
b	*Pogonus mannerheimi* Dej.			+										
b	*Pogonus maurusiacus* Mnnh							+						
b	*Pogonus cf. maurusiacus* Mnnh							+						
sa	*Pogonus parens* Tsch.							+						
	*Pogonus (Eosteropus)* sp.			+					+					
sa	*Pogonus montanus* Motsch.						+	+						
sa	*Pogonus cf. montanus* Motsch.		+											
sa	*Pogonus kokeili ssp. archangelicus* Popp.						+							
sa	*Pogonus tundrae* Tsch.		+	+			+	+						
sa	*Pogonus cf. tundrae* Tsch.			+				+						
sa	*Pogonus cf. abnormis* J.R.Sahlb.							+						
	*Pogonus (Petrophilus)* sp.			+				+	+	+				
sa	*Pogonus agonus* Horn.		+				+							
a	*Pogonus vermiculosus* Men.	+	+	+	+		+	+						
a	*Pogonus cf. cancellatus* Motsch.		+											
a	*Pogonus costatus* Men.		+	+	+		+	+						
a	*Pogonus sublaevis* J.R.Sahlb.		+	+			+	+						
sa	*Pogonus tareumiut* Ball.					+								
sa	*Pogonus cf. tareumiut* Ball.						+							
sa	*Pogonus theeli* Maekl.					+								
sa	*Pogonus cf. theeli* Maekl.						+							
sa	*Pogonus middendorfii* J.Sahlb.						+	+						
sa	*Pogonus cf. middendorffi* J.R.Sahlb.							+	+	+				
sa	*Pogonus ventricosus* Esch.			+			+	+					+	
sa	*Pogonus cf. ventricosus* Esch.		+	+									+	
sa	*Pogonus pinguedineus* Esch.	+				+								
sa	*Pogonus cf. pinguedineus* Esch.		+	+				+	+	+			+	
sa	*Pogonus cf. nigripalpis* Popp.	+		+										
sa	*Pogonus negligens* Sturm.			+			+	+		+		+	+	
sa	*Pogonus cf. negligens* Sturm		+							+				
sa	*Pogonus brevicornis* Kby.			+		+	+	+		+			+	
sa	*Pogonus cf. brevicornis* Kby		+						+					
sa	*Pogonus (Cryobius)* sp.	+	+	+	+		+	+	+	+	+		+	+
b	*Pogonus diligens* Sturm						+	+	+				+	
b	*Pogonus cf. diligens* Sturm								+	+				
b	*Pogonus cf. strenuus* Panz.									+				+
b	*Pogonus (Phonias)* sp.				+			+		+				
	*Pogonus* sp.		+	+					+	+			+	+
sa	*Stereocerus haematopus* Dej.		+	+			+	+		+				
sa	*Stereocerus cf. haematopus* Dej.							+						
sa	*Stereocerus rubripes* Motsch.							+						
sa	*Stereocerus cf. rubripes* Motsch.						+	+						
sa	*Stereocerus* sp.						+							
	*Platynus* sp.								+	+				
sa	*Agonum alpinum* Motsch.												+	
p	*Agonum cf. versutum* Sturm												+	
p	*Agonum ericeti* Panz.				+		+							
p	*Agonum micans* Nic.								+					
p	*Agonum cf. gracile* Gyll.						+							
	*Agonum (Europhilus)* sp.								+					
	*Agonum* sp.	+		+			+	+		+	+			
b	*Synuchus vivalis* Payk.			+										
sb	*Pseudotaphoxenus dauricus* F.-W.									+				
sa	*Amara quenseli* Shoenh.						+						+	
a	*Agonum glacialis* Mnnh.						+	+						
sa	*Agonum erratica* Duft.							+		+				
b	*Agonum minuta* Motsch.							+						
sa	*Agonum interstitialis* Dej.						+	+	+		+			
b	*Agonum brunnea* Gyll .			+				+		+			+	+
b	*Agonum cf. brunnea* Gyll.							+						
	*Agonum (Bradytus)* sp.								+					
	*Agonum (Celia)* sp.								+					
	*Agonum* sp.						+	+	+	+			+	
sa	*Curtonotus hyperboreus* Dej.								+					
a	*Curtonotus alpinus* Payk.		+	+		+	+	+		+				
a	*Curtonotus cf. alpinus* Payk.									+				
sa	*Curtonotus torridus* Panz.					+		+	+	+			+	
sa	*Curtonotus cf. torridus* Panz.		+					+	+	+				
sb	*Curtonotus dauricus* Motsch.									+				
	*Curtonotus* sp.		+	+				+	+	+	+			
sa	*Harpalus nigritarsis* C.R.Sahlb.							+	+	+			+	
sa	*Harpalus cf. nigritarsis* C.R.Sahlb.								+				+	
sb	*Harpalus cf. pulvinatus* Men												+	
	*Harpalus* sp.								+	+				
sa	*Dicheirotrichus mannerheimi* R. F. Sahlb.						+	+	+	+				
sb	*Cymindis mannerheimi* Gebl.									+		+	+	
sa	*Cymindis macularis* F.-W.								+	+				
b	*Cymindis cf. rivularis* Motsch.									+				
	*Cymindis* sp.								+	+				
	Carabidae indet.			+			+	+						
	Dytiscidae:													
	*Agabus (Gaurodytes)* sp		+	+	+		+	+					+	
	*Agabus* sp.							+	+	+			+	+
	*Hydroporus* sp.	+					+		+		+			
	Dytiscidae indet.						+	+						
	Gyrinidae:													
	*Gyrinus* sp.			+					+					
	Hydrophilidae:													
p	*Hydrobius fuscipes* L.			+				+	+					
p	*Helophorus cf. nubilis* F.	+												
sa	*Helophorus obscurellus* Popp.						+							
sa	*Helophorus cf. obscurellus* Popp.								+					
	*Helophorus* sp.		+				+	+	+	+	+		+	
	?*Helophorus* sp.							+						
	*Cercyon* sp.				+		+	+	+	+	+		+	
	Histeridae:													
	*Margarinotus* sp.								+					
	Catopidae:													
	*Catops* sp.		+	+		+	+	+	+		+			
	*Colon* sp.									+				
	Silphidae:													
sb	*Aclypaea sericea* Zoubk.								+					
sb	*Aclypaea bicarinata* Gebl.								+		+			
p	*Aclypaea opaca* L.						+	+	+	+	+		+	
	*Thanatophilus* sp.			+			+		+					
	Liodidae:													
	*Agathidium* sp.			+			+		+	+	+		+	
	*Anisotoma* sp.			+										
	*Liodes* sp.							+	+	+	+			+
	Staphylinidae:													
p	*Acidota crenata* Mnnh.										+			
p	*Acidota cf. cruentata* Mnnh.						+							
	*Acidota* sp.										+			
	*Olophrum* sp.			+			+		+					+
	Omaliinae gen. sp.	+	+	+				+	+	+	+		+	
a	*Tachinus cf. arcticus* Maekl.	+	+	+		+	+	+	+					
	Omaliinae gen. sp.													
	*Ocypus* sp.									+				
	Oxythelinae gen. sp.									+	+			
	*Tachinus* sp.			+	+			+	+	+			+	
	?*Mycetoporus* sp.								+					
	? *Philonthus* sp.		+											
	Tachyporinae gen. sp.			+							+			
	*Stenus* sp.			+			+	+	+					
	*Lathrobium* sp.			+			+				+			+
	Paederinae gen sp.			+					+		+			
	*Quedinus* sp.			+					+					+
p	*Scaphisoma* sp.						+							
	Staphylinidae indet.			+	+	+	+	+						
	Scarabaeidae:													
t	*Aphodius distinctus* Müll.								+					
t	*Acidota cf. distinctus* Müll.						+	+	+				+	
t	*Acidota cf. melanostictus* W.Schm.							+		+	+			
t	*Acidota cf. fossor* L.								+		+			
t	*Acidota cf. brevis* Er.								+		+			
t	*Acidota cf. rufipes* L.												+	
	*Acidota* sp.			+			+	+	+	+	+		+	
p	*Aegialia abdita* Nikritin							+	+	+	+		+	
	Helodidae:													
	*Cyphon* sp.							+			+			
	?*Cyphon* sp.													
	Dermestidae:													
	Dermestidae indet.								+					
	Byrrhidae:													
	*Byrrhus* sp.	+							+		+		+	
sb	*Porcinolus murinus* F.								+	+				
sa	*Morychus viridis* Kuzm. et Kor.			+			+	+			+			
sa	*Morychus cf. viridis* Kuzm. et. Kor.								+					
	*Morychus* sp.		+							+			+	
	*Simplocaria* sp.			+	+		+	+	+				+	
	*Curimopsis* sp.			+										
	Byrrhidae gen. sp.			+					+					
	Anobiidae:													
p	*Caenocara bovistae* Hoffm.			+			+							
	Heteroceridae:													
	*Heteroceris* sp.			+										
	Elateridae:													
p	*Hypnoidus cf. rivularis* Gyll.							+						
	*Hypnoidus* sp.			+						+				
	Nitidulidae:													
	Nitidulidae gen. indet.								+	+	+			
	Cryptophagidae:													
	Cryptophagidae indet.						+							
	Erotylidae:													
	Erotylidae indet.												+	
	Coccinellidae:													
b	*Scymnus* sp.									+				
sa	*Hippodamia arctica* Schneider									+				
b	*Coccinella trifasciata* L.							+						
b	*Coccinella cf. hieroglyphica* L.									+				
b	*Coccinella* sp.									+				
	Latridiidae:													
	Latridiidae gen.sp.	+									+			
	Oedemeridae:													
	Oedemeridae gen. sp.			+										
	Anthicidae:													
	Anthicidae gen.sp.										+			
	Tenebrionidae:													
sb	*Belopus* sp.								+					
	Chrysomelidae:													
	*Donacia* sp.	+					+							
sb	*Chrysolina perforata* Gebl.									+				
sb	*Chrysolina cf. perforata* Gebl.		+											
sb	*Chrysolina cf. aeruginosa* Fald.		+											
a	*Chrysolina cf. cavigera* J.R.Sahlb.		+											
a	*Chrysolina cf. subsulcata* Esch.		+	+										
sa	*Chrysolina septentrionalis* Men.		+											
sa	*Chrysolina cf. septentrionalis* Men							+						
p	*Chrysolina cf. graminis* L.								+					
	*Chrysolina* sp.		+	+			+	+	+	+				
a	*Chrysomela cf. taimyrensis* L. Medv.		+											
	*Chrysolina* sp.			+		+	+	+		+	+		+	
sb	*Colaphellus sophiae* Schall.								+	+	+			
p	*Hydrothassa hannoverana* F.												+	
	*Phaedon* sp.		+							+				
p	*Plagoiodera versicolora* Laich.		+						+					
	*Crosita* sp.							+	+					
	*Phratora* sp.			+										
	*Chalcoides* sp.								+					
	?*Chalcoides* sp.								+					
	?*Chaetocnema* sp.								+					
	*Altica* sp.			+										
	Alticinae gen. sp.			+										
	Chrysomelidae indet.			+		+								
	Erirhinidae:													
p	*Tournotaris bimaculatus* F.			+			+	+	+	+	+	+	+	+
b	*Tournotaris ochoticus* Kor.						+	+						
b	*Tournotaris cf. ochoticus* Kor.													+
p	*Notaris aethiops* F.						+	+		+	+			+
	*Notaris* sp.	+					+		+				+	
	Curculionidae:													
sb	*Otiorhynchus unctuosus* Germ.										+			
b	*Otiorhynchus politus* Gyll.									+	+	+		+
b	*Otiorhynchus cf. politus* Gyll.								+	+	+	+	+	+
sb	*Otiorhynchus wittmeri* Legalov									+				
sb	*Otiorhynchus cf. wittmeri* Legalov									+				
sa	*Otiorhynchus cf. arcticus* F.		+											
p	*Otiorhynchus ovatus* L.										+			
p	*Otiorhynchus cf. ovatus* L.										+			
	*Otiorhynchus* sp.						+		+	+	+		+	+
sa	*Sitona cf. ovipennis ssp. borealis* Kor.		+				+							
	*Sitona* sp.	+									+			
b	*Chlorophanus cf. sibiricus* Gyll.										+			
	*Chlorophanus* sp.						+							
?p	*Phyllobius cf. crassipes* Motsch et *Phyllobius maculicornis* Germ.			+										
	*Phyllobius* sp.						+	+	+	+	+		+	
	*Strophosoma* sp.							+						
sb	*Eusomus ovulum* Germ.													+
a-sb	*Coniocleonus ferrugineus* Fahr										+		+	+
a-sb	*Coniocleonus cf. ferrugineus* Fahr		+											
	*Coniocleonus* sp.		+							+				+
a-sb	*Stephanocleonus eruditus* Fast.						+							
	*Stephanocleonus* sp.										+		+	
sb	*Bothynoderes foveocollis* Gebl.										+			
	Cleoninae indet.					+	+	+	+	+			+	
p	*Hypera rumicus* L.							+						
p	*Hypera cf. ornata* Cap.		+				+	+						
p	*Hypera elongata* Pk.			+										
	*Hypera* sp.			+	+	+	+	+	+	+	+		+	+
sa	*Lepyrus nordenskjoldi* Faust.						+	+						
sa	*Lepyrus cf. nordenskjoldi* Faust		+	+										
sa	*Lepyrus cf. arcticus* Pk.	+												
	L. sp.					+	+	+					+	
b	*Trichalophus maeklini* Faust		+											
	*Trichalophus* sp.													+
p	*Phytobius cf. velaris* Gyll.		+		+									
b	*Callirus albosparsus* Boh.						+							
b	*Callirus* sp.										+			
b	*Pissodes* sp.							+			+			
	*Bagous* sp.						+			+				
	?*Limnobaris* sp.								+					
b	?*Magdalis* sp.												+	
b	*Rhyncholus ater* L.										+			
	*Anthonomus* sp.									+	+			
p	*Dorytomus cf. imbecillus* Faust							+						
	*Dorytomus* sp.							+		+	+			+
p	*Ceutorrhynchus cf. erysimi* F.						+							
	*Callirus* sp.						+	+					+	
p	*Isochnus saliceti* Müll	+												
sa	*Isochnus arcticus* Kor.			+				+						
	*Rhynchaenus* sp.		+		+				+	+	+			
	Curculionidae indet.						+	+						
	Brentidae:													
sa	*Hemitrichapion tschernovi* T.-M.			+	+		+	+						
	*Hemitrichapion* sp.													
p	*Mesotrichapion cf. punctirostre* Gyll.						+	+						
p	*Betulapion simile* Kby									+			+	
p	*Hemitrichapion cf. simile* Kby.			+										
	*Cyanapion* sp.									+				
	Brentidae gen.sp.	+	+	+			+	+		+	+		+	
	*Scolytidae*:													
b	*Phoelotribus spinulosus* Rey.							+	+		+		+	
b	*Polygraphus* sp.								+					

## Discussion

### Interpretation of the beetle communities

At first, these faunas suggest cooler than present climatic conditions, which is confirmed by the occurrence of sub-arctic species (*Pterostichus (Cryobius) cf. pinguedineus*, *Pterostichus ventricosus*, *Curtonotus torridus* and the arctic species (*Curtonotus alpinus*). This shows their southward distribution relative to their modern ranges.

As evidence of the lack of dense forest, is an absence of such typically boreal beetles as *Calathus micropterus*, *Pterostichus adstricus*, *Pterostichus oblongopunctatus* and others inhabiting the forest litter; at present they are widely distributed in the vast territories of West Siberia. Single boreal species are rare in the “mixed” faunas and are represented mainly by bark beetles (for example, *Phoelitribus spinulosus*, associated with spruce). The presence of sub-boreal beetles, inhabiting modern East-Siberian steppes (*Poecilus ravus*, *Poecilus hanhaicus*) and sub-alpine grasslands (*Cymindis mannerheimi*) could indicate open landscapes. An abundance of weevils of the genus *Otiorhynchus* may be explained by the wide distribution of herbal meadow vegetation. These faunas differ from contemporary insect steppe communities by the lack of darkling beetles, occurring in modern steppes and forest steppes (*Oodescelis polita*, *Crypticus quisquilius*, *Platyscelis hypolita*, *Opatrum riparium* etc.); it is possible that their absence was a result of cold climatic conditions.

At the same time, the presence of halophylous species, such as ground beetles of the genus *Pogonus*, darkling beetles of the genus *Belopus*, may indicate local soil salinity. At present these halophilic species are distributed southwards from 56–57°N and are very rare between 57–58°N, situated in sites with “mixed” faunas. Moreover, I am not aware of the presence of halophilic species of the genera Pogonus and Belopus in Central and East Siberia.

It may be assumed that such faunas inhabited open communities which can be defined as “cool grasslands” with a presence of rarefied forests or of single trees and local soil salinity. Similar conclusions have been drawn from palaeo-botanical data obtained at the same sites: they show a dominance of herbal vegetation with an abundance of cereals, wormwoods and chenopodiaceous plants.

The occurrence of some boreal faunas in single sites (Niznyaya Tavda, C14 27,400 ± 335 yr. BP) do not contradict the overall distribution of open landscapes, and show the presence of isolated patches of forest vegetation, like in modern forest steppes.

Therefore, entomological data show that at the time of the terminal phase of Middle Pleninglacial the following types of landscapes were distributed in the territories of the Ural Mountains and West Siberia: the northern part of the region north of 61°N was dominated by open landscapes similar to modern tundra, between 64 and 62°N - similar to forest tundra and between 59 and 57°N – non-analogue landscapes, which may be defined as “open grasslands” or savannas with a presence of rarefied forests.

The main influence on the natural ecosystems came from palaeo-environmental factors. The Middle Weichselian Interstadial was characterized by a continental and a cooler-than-present climate with low winter temperatures and a wide distribution of permafrost; the resulting development of large ice sheets caused a strong drying effect. Decreasing sea levels provided the opening of sea shelves and the connection between Europe and the British Isles, and the Beringian Bridge between Siberia and Alaska. Cold and dry climatic conditions reconstructed for main territories of Europe, even for the Mediterranean region (Adams and Faure 1997) may have caused a wide distribution of open landscapes such as tundra steppes of grasslands corresponding with the periglacial zone or “hyperzone” (Velichko 1973). Palaeoenvironmental reconstructions of “mixed” insect faunas from localities situated in the Ural Mountains and West Siberia show the presence of landscapes similar to savannas with rarefied woody vegetation. According to palaeo-botanical data (Stefanovsky et al. 2007), these “mixed” faunas correspond with open plant associations with an abundance of herbal vegetation.

It is necessary to define which factors might prevent the distribution of woods in the period of the Late Pleistocene studied. The main environmental factor is climate as a combination of thermal regime, precipitation, insulation, etc. At present I can suggest that severe climatic conditions similar to the palaeo-environment of the terminal phase end of the Middle Pleninglacial in the Central part of North Eurasia between 59° and 57°N are presented in the inner parts of Central and East Siberia. However, the modern conditions of cool and continental climate cannot avert the present distribution of woodland vegetation in this area. I suggest that not only climatic factor prevented of the distribution of woods in the central part of northern Eurasia between 59° and 57°N. Apart from climate, other factors might influence Pleistocene ecosystems; these factors may have impeded reforestation and stimulate the distribution of open landscapes.

The influence of mammoths and other large herbivorous mammals representing the “mammoth fauna” is probably large. It is known that vast areas of the continent were occupied by mammals belonging to the mammoth complex at that time (Markova et al. 2008).

### Evidence for the co-occurrence of insects with mega mammals of the “mammoth” fauna

Firstly, in many sites fossil insects were found along with mammoth remains (*Mammuthus primigenius*) and other large herbivorous mammals (teeth, tusks, fragments of cranium, etc.) (Borodin et al. 2001).

Secondly, in the majority of sites fragments of dung beetles of the genus *Aphodius*, were found which suggests the presence of mammoths and other large herbivorous mammals in the same landscapes (Sher and Kuzmina 2007).

According to the literature, mammoths and other mega mammals such as woolly rhinoceros (*Coelodonta antiquitatis*), giant deer (*Megaloceros giganteus*), reindeer (*Rangifer tarandus*), wild ox (*Ovibos moschatus*), primitive bison (*Bison priscus*)and some others may be considered as an additional factor, which influenced Late Pleistocene ecosystems (May 1993; Puchkov 2001).

Mammoths and other mammals were indicators of certain communities, and preserved specific ecosystems (Puchkov 2001):

1. Destruction of undergrowth and feeding impeded reforestation and might preserve herbal communities.

2. The hooves of mammoths destroyed the moss turf; as a result, moss cover disappeared in the territories of modern taiga and tundra zones, being replaced by mezo- and xerophylous herbal vegetation.

That is, mammoths and other mega mammals could rarefy forests and promote the distribution of zoogenic herbal vegetation consisting of cereals (Stuart and Hibbard 1986; Stuart 1991; May 1993; Puchkov 2001).

Consequently, Pleistocene forests were rare, and meadow and steppe plants were significant in the Siberian ecosystems.

I therefore suggest that the species composition of insects was affected by two important factors:

1. Cool and dry climate which caused low winter temperatures and a wide distribution of permafrost.

2. Pasture of large herbivorous mammals (mammoth and accompanying species) which caused the formation of «pasture» savannas with an abundance of herbal vegetation and rare forests.

### Do these factors define the composition of insect complexes as “mixed” faunas at 59°–56°N?

Firstly, cool and dry climate may cause a southward advance of arctic and sub-arctic species (*Diacheila polita*, *Curtonotus alpinus*, *Curtonotus torridus*, *Pterostichus (Cryobius)* spp.). As such, the warming and drying of local habitats (such as slopes with a southern exposition) in dry and cold climatic conditions and their subsequent salinity may cause the occurrence of some halophilic beetles.

Secondly, the pasture of mammoths and other mega mammals may cause the distribution of grasslands with a dominance of cereals and an abundance of weevils of the genus *Otiorhynchus*. The presence of sub-boreal steppe and sub-alpine species (*Poecilus ravus*, *Cymindis mannerheimi*, *Chrysolina perforata*) may have been caused by both environmental conditions and pasturable load. Rarefaction of woods may explain the lack of species inhabiting forest litter (*Calathus micropterus*, *Pterostichus oblongopunctatus* etc.); presence of single trees - occurrences of xylophagous beetles (bark beetle *Phoelotribus spinulosus* etc.). The fertilization of the soil may have caused the occurrence of coprophagous beetles (dung beetles of the genera *Aphodius*).

A combination of these factors may have caused the distribution of several landscapes.

In the central and northern parts of the region north of 59°N, the cold climate and corresponding mammoth pasture formed communities similar to modern tundra and forest tundra. South of 59°N and up to 57°N, specific landscapes and according insect faunas were formed. These conclusions do not contradict literature data on the palaeo-geography of that period (Arkhipov and Volkova 1994; Volkova et al. 2005; Astakhov 2009).

It may be assumed, that ground beetles of the species *Carabus sibiricus*, *Poecilus ravus*, *Pterostichus pinguedineus*, *Cymindis mannerheimi* and others have been widely distributed in the territories of the central part of Northern Eurasia, so that these insects may form an integral part of the landscapes containing the “mammoth faunas”.

### Factors leading to the disappearance of the “mammoth faunas”

At the beginning of the Holocene (10,000 yr. BP) in the Northern Hemisphere significant climatic changes took place, modifying all natural communities, and the final degradation of “mammoth faunas” took place. The largest mammals, mammoth (*Mammuthus primigenius*), woolly rhinoceros (*Coelodonta antiquitatis*), and giant deer (*Megaloceros giganteus*), having the greatest effect on terrestrial ecosystems, died out about 10–8,000 years ago, and the ranges of other species, such as reindeer (*Rangifer tarandus*), musk ox (*Ovibos moschatus*)) shifted either northwards to the tundra and forest tundra, or southwards, to the steppes, such as the saiga antelope (*Saiga tatarica*).

The subsequent Early Holocene warming and humidification of climate, the extinction of mammoths and its consequent failing of the “pasture load” caused the reforestation and water logging of vast territories, the formation of the boreal belt, the transformation of the flora and fauna, and the wide distribution of conifer forests.

Therefore, climatic changes and the extinction of the large mammals happening between the Pleistocene and Holocene caused the disappearance of the “mixed” or non-analogue insect faunas. However, the insect species did not die out, but only changed the location of their ranges. At that time the “mixed” insect faunas disintegrated into groups of single species, shifting their ranges northwards (*Curtonotus alpinus*, *Pterostichus (Cryobius)* spp.), southwards (*Pogonus* spp., *Cymindis mannerheimi*) or eastwards (*Poecilus (Derus) hanhaicus*, *Poecilus (Derus) ravus*, *Poecilus (Derus) major*, *Pseudotahoxenus dauricus*, *Amara minuta*). These species only left the territories studied but could survive these environmental changes in other regions of northern Eurasia, such as Mongolia, Eastern Siberia, or the Pamir Mountains, where environmental conditions are more compatible to their ecological requirements.

### Comparison with other regions of North Eurasia

The “mixed” or non-analogue faunas of the central part of North Eurasia were compared with synchronous insect faunas as described for East Siberia (Sher et al. 2005, Kuzmina and Sher 2006; Sher and Kuzmina, 2007). Significant differences between these regional faunas were found. Firstly, in the Late Quaternary insect complexes of Northeastern Siberia with remains of the pill beetle *Morychus viridis* were found in large quantities. Moreover Sher and Kuzmina claimed that *Morychus viridis* is “… a real symbol of the Pleistocene biota in Northeastern Siberia” (Sher and Kuzmina 2007, p. 105). Remains of *Morychus* similar to *Morychus viridis* were found in “mixed” faunas from the Ural Mountains and West Siberia, although these insects were not so numerous here. The MIS 3 insect assemblages of the study area characterized by an abundance of fragments of weevils of the genus *Otiorhynchus* and morphologically similar to *Otiorhynchus politus* did not occur in the East Siberian sub-fossil insect faunas. The steppe assemblages of fossil insects from East Siberia belong to species, which are not found in the “mixed” insect faunas of the Ural Mountains and West Siberia, such as weevils of the genus *Stepanocleonus* (*Stepanocleonus eruditus*, *Stepanocleonus fossulatus*), *Poecilus nearcticus*, *Harpalus vittatus*. Tundra steppe beetles, such as *Troglocollops arcticus*, and *Galeruca interrupta circumdata* were not found in the insect assemblages of the Ural Mountains and West Siberia. An important feature of these “mixed” faunas of the Central part of North Eurasia is the presence of halophilic beetles which indicates local soil salinity, which may be explained by a strong aridity of the climate and by an external biogenic influence (pasture load) on the landscapes. No halophilic insects were found in fossil insect assemblages in Central and East Siberia. However, in East Siberian MIS 3 faunas remains of dung beetles of the genus *Aphodius* were found, which are considered to indicate the presence of herbivorous mammals (Sher and Kuzmina 2007).

Sub-fossil insect assemblages from Northeastern Siberia may reflect the existence of tundra steppe landscapes which have no analogue among modern ecosystems (Sher et al. 2005). The climate forming these communities can be considered as a main factor, but by cutting and trampling of grasses herbivores including large mammals made their own contribution to the formation of these ecosystems. For a long time pasture load allowed the perpetuation of grazing ecosystems (Zimov et al. 1995, Sher et al. 2005).

Insect faunas at the end of the Middle Pleninglacial in Western Europe (Bos et al. 2004) differ strongly from our faunas by the lack of steppe, by the absence of halophilic species and by the occurrence of the pill beetle of the genus *Morychus*. Weevils of the genus *Otiorhynchus* from European sites belong to *Otiorhynchus dubius*, which is not found in West Siberian faunas.

It is possible that these faunas, belonging to the “mixed” type, were distributed mainly in the Central part of North Eurasia (including West Siberia and the Ural Mountains) during the Late Pleistocene (MIS 4-MIS 2). So, similar faunas were found in the Gornova site, situated in the South Ural Mountains, near Ufa city (data given by F.G.Bidashko (Kazakhstan)). These assemblages are characterized by abundance of remains of the genus *Otiorhynchus* (similar to *Otiorhynchus politus*), the presence of *Poecilus ravus*, *Pogonus* spp., *Belopus* spp. and other species, with the presence of some endemic forms (*Nedria uralensis*).

## Conclusions

1. Sub-fossil insect assemblages allow us to reconstruct several elements of the natural zonality which existed in the central part of Northern Eurasia during the terminal phase of the Middle Pleninglacial (MIS 3). In the northern and central parts of the region north of 59°N, the cold climate and the corresponding mammoth pasture formed communities similar to modern tundra and forest tundra. In the southern part of the study area between 57° and 59°N, specific landscapes and corresponding insect faunas formed, known as “mammoth savannas”.

2. Insect faunas of a “mixed” type of the Ural Mountains and West Siberia differ from East Siberian sub-fossil insect assemblages found in synchronous layers with the presence of numerous fragments of weevils *Otiorhynchus* which are morphologically similar to *Otiorhynchus politus*, as well as the halophilic beetles of the genera *Pogonus* and *Belopus*. Steppe beetles, such as weevils of the genus *Stepanocleonus* did not establish assemblages in West Siberia. Significant differences between insect assemblages from the central part of northern Eurasia and Western Europe were marked too. These faunas cannot be identified both as forest tundra nor tundra steppe and differ even from modern insect communities of East Siberia relict tundra steppes.

3. The species composition of insect complexes was determined not only by climate, but by pasture pressure of mammoths and other herbivorous mammals as well. A pasture load occurred in all territories of the Ural Mountains and West Siberia, but is defined differently in different parts of the study area. In the central and northern parts of the region north of 59°N, a combination of these factors formed communities similar to modern tundra and forest tundra in accordance to the southward advance of arctic and sub-arctic insect complexes relative to contemporary faunas. In those territories lying during the terminal phase of MIS 3 between 59° and 57°N insect faunas existed without any analogues among modern insect complexes and included sub-arctic, sub-boreal steppe species, halophilic insects and weevils of the genus *Otiorhynchus* and similar to *Otiorhynchus politus*.
